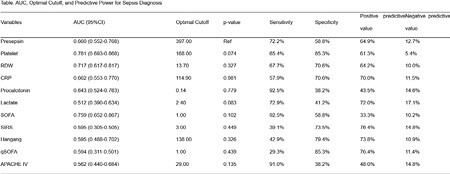# 509 Comparative Analysis of Presepsin and Other Biomarkers in Sepsis Diagnosis Among Burn Patients

**DOI:** 10.1093/jbcr/irae036.144

**Published:** 2024-04-17

**Authors:** Dohern Kym

**Affiliations:** Hangang Sacred Heart Hospital, Hallym University Medical Center, Seoul, Seoul-t'ukpyolsi

## Abstract

**Introduction:**

Sepsis and septic shock are life-threatening conditions that pose significant challenges in the medical field, particularly among burn patients. Early and accurate diagnosis is crucial for effective treatment and improved patient outcomes. This study aims to evaluate the diagnostic and prognostic utility of presepsin, a novel biomarker, in comparison to other established biomarkers like procalcitonin, Red Cell Distribution Width (RDW), and platelet count among burn patients suspected of infection.

**Methods:**

A prospective cohort study was conducted, involving 167 patients admitted to the Burn Intensive Care Unit (BICU) with suspected infection. Blood samples were collected within 24 hours of admission for the measurement of presepsin, procalcitonin, RDW, and platelet count. Patients were categorized based on the diagnosis and progression of sepsis and septic shock according to the Sepsis-3 criteria. The study employed logistic regression models to assess the predictive power of each biomarker.

**Results:**

Out of the 167 patients, 133 were diagnosed with sepsis, and the overall mortality rate was 38.9%. Presepsin demonstrated moderate predictive power for sepsis with an Area Under the Curve (AUC) of 0.660. However, it was not statistically significant for predicting mortality. In contrast, RDW and platelet count showed higher predictive values with AUCs of 0.780 and 0.760, respectively.

**Conclusions:**

While presepsin showed limited utility in the diagnosis and prognosis of sepsis and septic shock, other biomarkers like RDW and platelet count exhibited stronger predictive capabilities. These findings suggest that a multi-biomarker approach could enhance the early diagnosis and management of sepsis and septic shock in burn patients.

**Applicability of Research to Practice:**

The study's findings have significant implications for clinical practice. Understanding the predictive potential of various biomarkers can guide clinicians in the early diagnosis and effective management of sepsis and septic shock, particularly in burn patients.